# Cutaneous adverse reaction during lithium treatment: a case report and updated systematic review with meta-analysis

**DOI:** 10.1186/s40345-017-0091-7

**Published:** 2017-07-04

**Authors:** Martina Pinna, Mirko Manchia, Sergio Puddu, Giampaolo Minnai, Leonardo Tondo, Piergiorgio Salis

**Affiliations:** 1 Psychiatry Unit, San Martino Hospital-Regional Health Agency, Oristano, Sardinia Italy; 2 Section of Psychiatry, Department of Medical Science and Public Health, University of Cagliari, Via Liguria, 13, 09127 Cagliari, Sardinia Italy; 3 Department of Pharmacology, Dalhousie University, Halifax, NS Canada; 4 Regional Health Agency, National Health System, Oristano, Sardinia Italy; 5 Department of Psychiatry, Harvard Medical School, Boston, MA USA; 6 The International Consortium for Mood & Psychotic Disorders Research, McLean Hospital, Belmont, MA USA; 7 Lucio Bini Mood Disorders Centers, Cagliari and Rome, Italy

**Keywords:** Skin disorders, Bipolar disorder, Long-term treatment

## Abstract

**Objectives:**

To present a new case of adverse cutaneous reaction during lithium treatment and to update the systematic review and meta-analysis of the incidence of this adverse reaction.

**Methods:**

We conducted a systematic search (performed in September 2016) for peer-reviewed articles in English indexed in Medline (2011-present). Meta-analytical estimates were obtained using the “Metafor” package.

**Case presentation:**

Ms. H., a 31-year-old Caucasian woman with BD1, was admitted to the inpatient unit for a full-blown psychotic episode and treated with carbamazepine 400 mg q.d., lithium carbonate 450 mg q.d., and risperidone 4 mg q.d. with clinical improvement. After 12 days from the start of psychopharmacological treatment, she manifested a cutaneous reaction that motivated the stop of carbamazepine treatment, as well as the increase in lithium carbonate dose (750 mg q.d.). Risperidone dose remained unvaried. Since the skin lesion persisted after 8 days from withdrawal of carbamazepine, the private practitioner stopped also lithium carbonate treatment (de-challenge), maintaining risperidone treatment. The cutaneous reaction resolved spontaneously after six days from withdrawal of lithium carbonate. Subsequently, the worsening of psychopathological conditions motivated a new admission during which lithium carbonate was reintroduced (16 days after its suspension) (re-challenge). On the following day, we observed an itching erythematous maculopapular rash involving the trunk, the four limbs, and the oral mucosa.

**Conclusions:**

Our case of an erythematous maculopapular rash during lithium treatment was the first to present a challenge–de-challenge–re-challenge sequence that suggests causality. Although meta-analysis does not point to an increased rate of adverse skin reaction during lithium treatment, clinicians should not neglect to monitor cutaneous symptoms during lithium treatment.

## Background

Lithium remains the mainstay for the treatment and prevention of mood episodes in bipolar disorder (BD) (Yatham et al. 2013). Its prophylactic effectiveness has been demonstrated by naturalistic observational studies which showed that approximately one-third of lithium-treated BD patients achieve full symptomatic remission (Garnham et al. 2007; Baldessarini and Tondo 2000), and that this pattern of clinical response remains stable in the long-term (Berghofer et al. 2013).

However, the effectiveness of lithium should be weighed against its side effect profile that might limit its acceptability by patients (McKnight et al. 2012). Indeed, it is well established that lithium treatment can impact kidney, thyroid, and parathyroid function (McKnight et al. 2012). A degree of uncertainty remains, however, on whether lithium, during short- and long-term treatment, is associated with the manifestation of cutaneous adverse reactions. Although quantitative meta-analytical estimates did not show an increased rate of skin disorders in lithium-treated patients compared to those given placebo (McKnight et al. 2012), there is substantial anecdotic evidence supporting this association. Indeed, some authors indicated that the rate of cutaneous adverse reactions during lithium maintenance therapy might be as high as 45% (Jafferany 2008). Here, firstly we presented a new case of adverse cutaneous reaction during lithium treatment, confirmed by a re-challenge of the drug after its discontinuation. We then updated the study of McKnight et al. (2012) which systematically reviewed and performed data synthesis of pertinent literature published until 2010 (McKnight et al. 2012).

## Case presentation

Ms. H., a 31-year-old Caucasian woman with BD1, was voluntarily admitted to the psychiatric ward of the San Martino Hospital of Oristano, Sardinia, Italy for a manic episode with mood-congruent psychotic features according to the Diagnostic and Statistical Manual of Mental Disorders-5 (DSM-5) criteria.

The family history of Ms. H. was positive for mood disorders: her father was diagnosed with BD1 and her paternal grandmother with mood disorder not otherwise specified (NOS). Her premorbid personality had been characterized by cyclothymic temperament and rejection sensitivity. Her first illness episode presented at 21 years in concomitance with the end of a romantic relationship. Specifically, she developed depressed mood, social withdrawal, and persecutory delusion for which she was treated with unspecified psychopharmacological treatment. At the age of 24, during a euthymic phase, and in the absence of pharmacological treatment, she started presenting somatic symptoms (abdominal pain, change in bowel habits, and bloating) after a surgical operation for ovarian cyst removal. Around the same time, she experienced marked anxiety, ruminations, and social discomfort with subsequent social withdrawal. Treatment with selective serotonin reuptake inhibitors (SSRI) was started with clinical remission.

Although in the following years the patient had a good social and work functioning, several residual psychopathological symptoms remained, including mood fluctuations, hypochondriac thoughts and behaviors, marked anxiety, and dietary restraint with weight loss aimed at facilitating control of bowel function. The absence of a complete clinical remission was partially due to the lack of compliance to clinical monitoring and psychopharmacological treatment. The progressive increase in the severity of these psychopathological symptoms led to manifestation of a full-blown psychotic episode in early 2016. This clinical picture motivated a 1-week admission at the end of March 2016 at the acute inpatient psychiatric unit in Pisa, Italy, where she was started on carbamazepine 400 mg q.d., lithium carbonate 450 mg q.d., and risperidone 4 mg q.d., which led to substantial clinical improvement. Laboratory workup, including thyroid function parameters, was normal. After discharge from hospital, she moved to Sardinia and was seen by a private practitioner for follow-up visits. After 12 days from the start of psychopharmacological treatment, she manifested a cutaneous reaction that motivated the stop of carbamazepine treatment, as well as the increase in lithium carbonate dose (750 mg q.d.) (challenge phase). Risperidone dose remained unvaried. Since the skin lesion persisted after 8 days from withdrawal of carbamazepine, the private practitioner stopped also lithium carbonate treatment (de-challenge), maintaining risperidone treatment. The cutaneous reaction resolved spontaneously after 6 days from withdrawal of lithium carbonate. On May 2016 after the manifestation of marked anxiety, flight of ideas, pressured speech, decreased ability to concentrate, insomnia, and persecutory delusions, she came to our attention and was admitted at our unit where lithium carbonate was reintroduced (16 days after its suspension) (re-challenge). On the following day, we observed an itching erythematous maculopapular rash involving the trunk, the four limbs, and the oral mucosa. The dermatologist consultation indicated the presence of: “a drug-induced toxic reaction, characterized morphologically by eruption of a large number of erythematous macules of varying size, mostly with papules, which became confluent and itching. It is observable also a palmoplantar eruption of pale erythematous macular lesions with scarlatiniform characteristics”. Lithium carbonate was immediately stopped with resolution of the skin lesion on the subsequent day. This severe adverse reaction was described and sent to the Sardinian Regional Centre of Pharmacovigilance. Their assessment with the Naranjo algorithm (Naranjo et al. 1981) classified the relationship between the cutaneous adverse reaction and the use of lithium as “probable.”

## Systematic review and meta-analysis

To update the study of McKnight et al. (2012), we conducted a systematic search (performed in September 2016) for peer-reviewed articles in English indexed in Medline (2011-present). The terms used for the electronic database search were: lithium AND (skin OR dermatology OR cutaneous side effects OR psoriasis OR rash OR Stevens-Johnson syndrome). Studies were included if they reported on adverse cutaneous reaction during lithium treatment. Data on cutaneous adverse reactions in published or unpublished randomized clinical trials (RCTs) since 2011, comparing lithium (as monotherapy or add-on) with placebo or other pharmacological treatments, were extracted from ClinicalTrials.gov, which is a database of publicly and privately supported clinical studies of human participants conducted around the world (www.ClinicalTrials.gov). Meta-analytical estimates were obtained using the “Metafor” package (Viechtbauer 2010). Since adverse cutaneous reactions in the included clinical trials comparing lithium with placebo were very rare, we used the Peto odds ratio (OR) fixed-effect method.

**Fig. 1:**
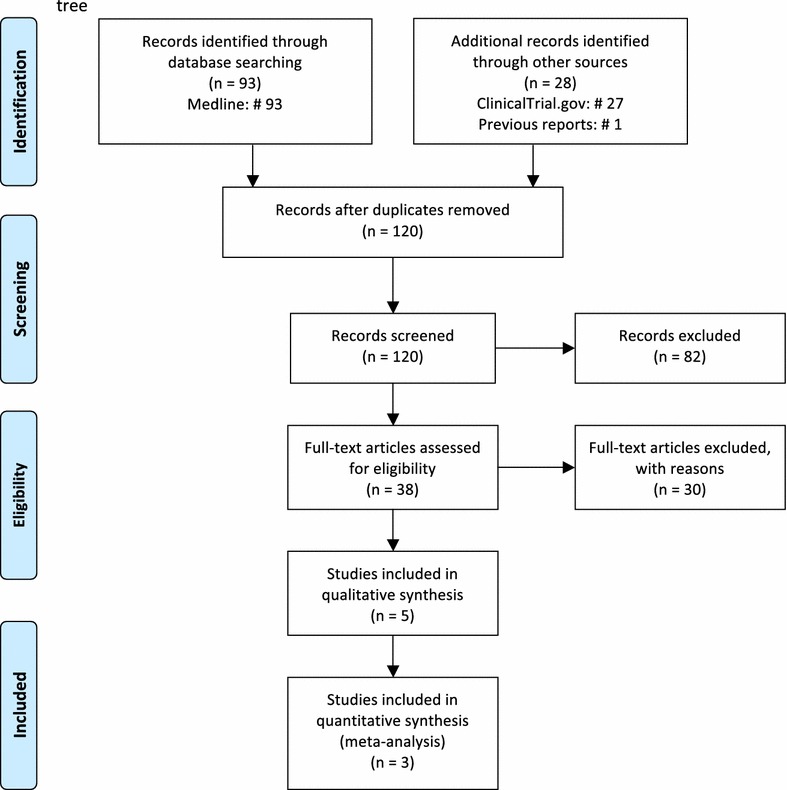
Preferred reporting items for systematic reviews and meta-analyses (PRISMA) tree

Our systematic search found 93 studies indexed in Medline. We also found 28 clinical trials including one previous study examined in the meta-analysis of McKnight et al. (2012). The review process is summarized in Fig. 1 according to the preferred reporting items for systematic reviews and meta-analyses (PRISMA) (Moher et al. 2009). Five of the studies indexed in Medline met inclusion criteria for the qualitative synthesis: four were case reports and one was an observational case/control study (Table 1). The four case reports presented a varying spectrum of skin disorders associated with lithium treatment. Specifically, Maejima et al. (2012) reported on a 55-year-old male affected by psychosis who developed psoriasis verrucosa, a rare and atypical form of psoriasis. Although the patient improved after treatment with adalimumab, a tumor necrosis factor-alpha (TNF-α) inhibitor, it was not specified whether an attempt was made to discontinue lithium in order to observe a decrease in the severity of the lesions.

**Table 1:** Cutaneous adverse reactions during lithium treatment: case reports and observational studies

Study								
Case reports	Number of patients	Gender	Age (years)	Diagnosis	Duration of lithium treatment	Adverse cutaneous reaction	C–D–R	Medical comorbidities
Maejima et al. (2012)	1	Male	55	Psychosis	NA	Psoriasis	NA	None
Wang and Yang (2013)	1	Female	37	BD1	7 days	Non-itching, erythematous, maculopapular rash over 70% of total body surface area	C–D	None
Scarfi and Arunachalam (2013)	1	Female	40	BD	4 months	Facial acne	C–D	None
Bugueno et al. (2013)	1	Female	25	BD	1 month	Oral lichenoid lesion	C–D	ADHD, sickle cell trait

Observational studies	Method	Diagnosis	Duration of lithium treatment	Number of patients	Outcome measured	Result
Spoendlin et al. (2014)	Matched case control study	Affective disorders, schizophrenia	>180 days	53,927	Incidence of rosacea	Lower rate of rosacea in long-term lithium-treated patients

*BD* bipolar disorder, *BD1* bipolar disorder type 1, *NA*: not available, *C–D–R* challenge–de-challenge–re-challenge, *C–D* challenge–de-challenge

The case described by Wang and Yang (2013) concerned a 37-year-old BD1 woman who presented a non-itching, erythematous, maculopapular rash after the start of therapy with lithium 900 mg q.d. which resolved spontaneously after its discontinuation. Further, Scarfi and Arunachalam (2013) reported on the manifestation of facial acne in a 40-year-old woman after 4 months of treatment. Again, the skin lesion completely resolved after treatment discontinuation. Finally, Bugueno et al. (2013) described the onset of an oral lichenoid mucosal lesion in concomitance with the initiation of the treatment in a 25-year-old BD female patient. Complete resolution of the skin lesion was observed after discontinuation of lithium. The large observational case-control study by Spoendlin et al. (2014) interestingly found that lithium treatment was associated with a decreased risk of rosacea.

Data on cutaneous adverse reactions were extracted for meta-analysis from three clinical trials: Goodwin et al. (2004), Geller et al. (2012), and NTC01189812 (Table 2). The first study was also included in the meta-analysis of McKnight et al. (2012). Meta-analysis did not show a significant difference in the rate of cutaneous adverse reaction between lithium- and placebo-treated patients (Peto OR 1.14, 95% [Confidence Interval (CI) 0.44–2.94], *P* = 0.78; heterogeneity *Q* = 0.92, *P* = 0.34), as well as between those treated with lithium or with other treatments (OR 0.61, [95% CI 0.34–1.11], *P* = 0.11; heterogeneity *Q* = 0.91, *P* = 0.63).

**Table 2:** Cutaneous adverse reactions during lithium treatment: randomized clinical trials

Study	Number of patients	Diagnosis	Duration of lithium treatment	Outcome measured	Follow-up	Results
Goodwin et al. (2004)	167 (166 completed) lithium 280 (227 completed) lamotrigine 191 (190 completed) placebo	BD1	NA	Self-reported incidence of new-onset skin disorders during treatment	18-months	Lithium: 5% Lamotrigine: 7% Placebo: 5%
Geller et al. (2012)	89 risperidone 90 (84 completed) lithium 100 (97 completed) valproic acid	BD1	NA	Systematic structured assessment of side effects	8 weeks	Lithium: 6% Risperidone: 6.7% Valproic acid: 13.4%
NCT01189812	40 citalopram + placebo 40 citalopram + lithium	MDD, Dysthymia DNOS, BPD	4 weeks	Self-reported incidence of new-onset skin disorders during treatment	4 weeks	Citalopram + lithium: 2.5% Citalopram + placebo: 2.5%

*NA* not available, *BD* bipolar disorder, *BD1* bipolar disorder type 1, *MDD* major depressive disorder, *DNOS* depression not otherwise specified, *BPD* borderline personality disorder

## Discussion and conclusion

We presented a case of a diffuse erythematous maculopapular rash during lithium treatment in a 31-year-old woman with BD1. This observation adds to the limited anecdotal literature on adverse cutaneous reaction during lithium treatment. The manifestation of the lesion appeared similar to that described in previous reports (Swartz and Holkesvick 1981; Wang and Yang 2013). However, differently from most of previously published reports, our clinical case strongly suggests causality between the use of lithium and the onset of the skin reaction, particularly in light of the reoccurrence of the maculopapular rash following drug re-challenge.

One could argue that the use of carbamazepine could be related to the manifestation of the skin reaction. However, the maculopapular rash persisted 8 days after discontinuation of carbamazepine and it is unlikely that the drug might have remained in the bloodstream for such a time given that its half-life should have been around 10–20 h after 8 weeks of treatment (Bertilsson 1978). Further, cross-reactivity, which is common for adverse skin reactions with carbamazepine and chemically related drugs, should be excluded since lithium does not have any similarity with the aromatic structure of carbamazepine.

One final comment is needed on the findings of our meta-analysis. We confirmed that the evidence so far available does not point to an association between lithium treatment and cutaneous adverse reaction. These findings should be reconciled, however, with the recurrent manifestation of these events in lithium-treated patients. One possible explanation is that the incidence rate of these events is quite heterogeneous, with estimates ranging from 3.4 to 45% (Jafferany 2008). Therefore, the probability of observing an adverse cutaneous reaction during lithium treatment depends on the design of the clinical trial (duration of follow-up, inclusion criteria, method of assessment of adverse reactions) as well as on the characteristics of the patients included (type of diagnosis, presence and duration of lithium treatment before the start of the clinical trial, pre-existing allergic diathesis, history of psoriasis). It is conceivable that large registry study might be more suitable to the task of quantifying the incidence of this adverse reaction of lithium.

In conclusion, we presented a case of an erythematous maculopapular rash during lithium treatment. Although this type of skin reaction has been reported previously in the literature, our case is the first to present a challenge–de-challenge–re-challenge sequence that suggests causality. Although meta-analysis does not point to an increased rate of adverse skin reaction during lithium treatment, clinicians should not neglect to monitor cutaneous symptoms, particularly in BD patients at high risk, such as those with an allergic diathesis.

## Abbreviations

BD: bipolar disorder
BD1: bipolar disorder type 1
DSM-5: diagnostic and statistical manual of mental disorders-5
SSRI: selective serotonin reuptake inhibitors
OR: odds ratio
CI: confidence interval
RCTs: randomized clinical trials
